# Mitochondrial unfolded protein response, mitophagy and other mitochondrial quality control mechanisms in heart disease and aged heart

**DOI:** 10.3325/cmj.2020.61.126

**Published:** 2020-04

**Authors:** Tomo Svaguša, Mislav Martinić, Matea Martinić, Lucija Kovačević, Ana Šepac, Davor Miličić, Joško Bulum, Boris Starčević, Maja Sirotković-Skerlev, Fran Seiwerth, Ana Kulić, Filip Sedlić

**Affiliations:** 1 Department of Cardiovascular Diseases, University of Zagreb School of Medicine, Clinical Hospital Dubrava, Zagreb, Croatia; 2 University of Zagreb School of Medicine, Zagreb, Croatia; 3 Department of Diagnostic and Interventional Radiology, University Hospital Center Zagreb, Zagreb, Croatia; 4 Department of Pathology, University of Zagreb School of Medicine, Zagreb, Croatia; 5 Department of Cardiovascular Diseases, University of Zagreb School of Medicine, University Hospital Center Zagreb, Zagreb, Croatia; 6 Department of Pathophysiology, University of Zagreb School of Medicine, Zagreb, Croatia; 7 Department for Respiratory Diseases Jordanovac, University of Zagreb School of Medicine, University Hospital Centre Zagreb, Zagreb, Croatia; 8 Department of Oncology, Division of Pathophysiology and Experimental Oncology, University Hospital Center Zagreb, Zagreb, Croatia

## Abstract

Mitochondria are involved in crucial homeostatic processes in the cell: the production of adenosine triphosphate and reactive oxygen species, and the release of pro-apoptotic molecules. Thus, cell survival depends on the maintenance of proper mitochondrial function by mitochondrial quality control. The most important mitochondrial quality control mechanisms are mitochondrial unfolded protein response, mitophagy, biogenesis, and fusion-fission dynamics. This review deals with mitochondrial quality control in heart diseases, especially myocardial infarction and heart failure. Some previous studies have demonstrated that the activation of mitochondrial quality control mechanisms may be beneficial for the heart, while others have shown that it may lead to heart damage. Our aim was to describe the mechanisms by which mitochondrial quality control contributes to heart protection or damage and to provide evidence that may resolve the seemingly contradictory results from the previous studies.

Mitochondria are involved in adenosine triphosphate (ATP) generation, biosynthetic processes and redox homeostasis. However, dysfunctional mitochondria can become a source of endogenous noxious stimuli that can severely damage the cells, such as the overproduction of reactive oxygen species (ROS), cellular calcium overload, opening of mitochondrial permeability transition pore (mPTP), and release of pro-apoptotic signals (1,2). Thus, it is of vital importance to maintain mitochondrial function by (intra)mitochondrial quality control (MQC) mechanisms. MQC either repairs the damaged mitochondria by restoring or destroying impaired proteins through the activation of mitochondrial unfolded protein response (UPRmt), or removes mitochondria damaged beyond repair by mitophagy (3). Mitophagy is closely balanced with mitochondrial biogenesis to maintain total mitochondrial mass. Rapid changes in fusion and fission of mitochondria are associated with ROS generation and apoptosis, but are also interconnected with other MQC mechanisms (4).

Cardiovascular diseases, especially acute myocardial infarction (MI) and chronic heart failure (HF), account for numerous deaths and severely undermine the quality of life (5,6). A crucial etiological factor in these diseases is mitochondrial dysfunction (7). The aim of this article is to review the present data on underlying mechanisms of heart disease, especially HF and MI, mediated by improper MQC functioning. While the broad scientific community recognizes MQC as a beneficial homeostatic mechanism, numerous studies demonstrate its opposite effects on cardiac diseases. Some studies report a cardioprotective role of MQC, while others show its negative effects in major heart disease. Here, we will try to provide a plausible explanation of such discrepancies. The article addresses major components of MQC in heart disease, including UPRmt, mitophagy, mitochondrial biogenesis, and mitochondrial fusion-fission, as well as MQC in the aged heart.

## Basic mechanisms of injury in myocardial infarction and heart failure

The common noxious stimuli in MI and HF are excessive ROS generation and mitochondrial calcium overload (1,2,8,9). In both diseases, ROS and mitochondrial calcium overload induce opening of the mPTP, which ultimately leads to apoptotic or necrotic cell death (1,2,10). Mitochondrial permeability transition pore opening initiates the events that lead to the release of intramitochondrial proapoptotic factors, including cytochrome c, diablo IAP-binding mitochondrial protein, HTRA serine peptidase 2 [OMI/HTRA2], apoptosis-inducing factor, and endonuclease G (11). It is believed that a less extensive mPTP opening results in apoptosis, mostly in the periphery of MI (12). More extensive mPTP opening in the center of MI leads to necrotic cell death, possibly due to severe ATP depletion and the inability to complete the energy-dependent process of apoptosis (12). A major role in the pathogenesis of heart injury is played by the substrates used for energy metabolism. For example, the use of fatty acids can enhance ROS generation and thereby damage cardiomyocytes (13).

## Mitochondrial unfolded protein response

**Figure 1:**
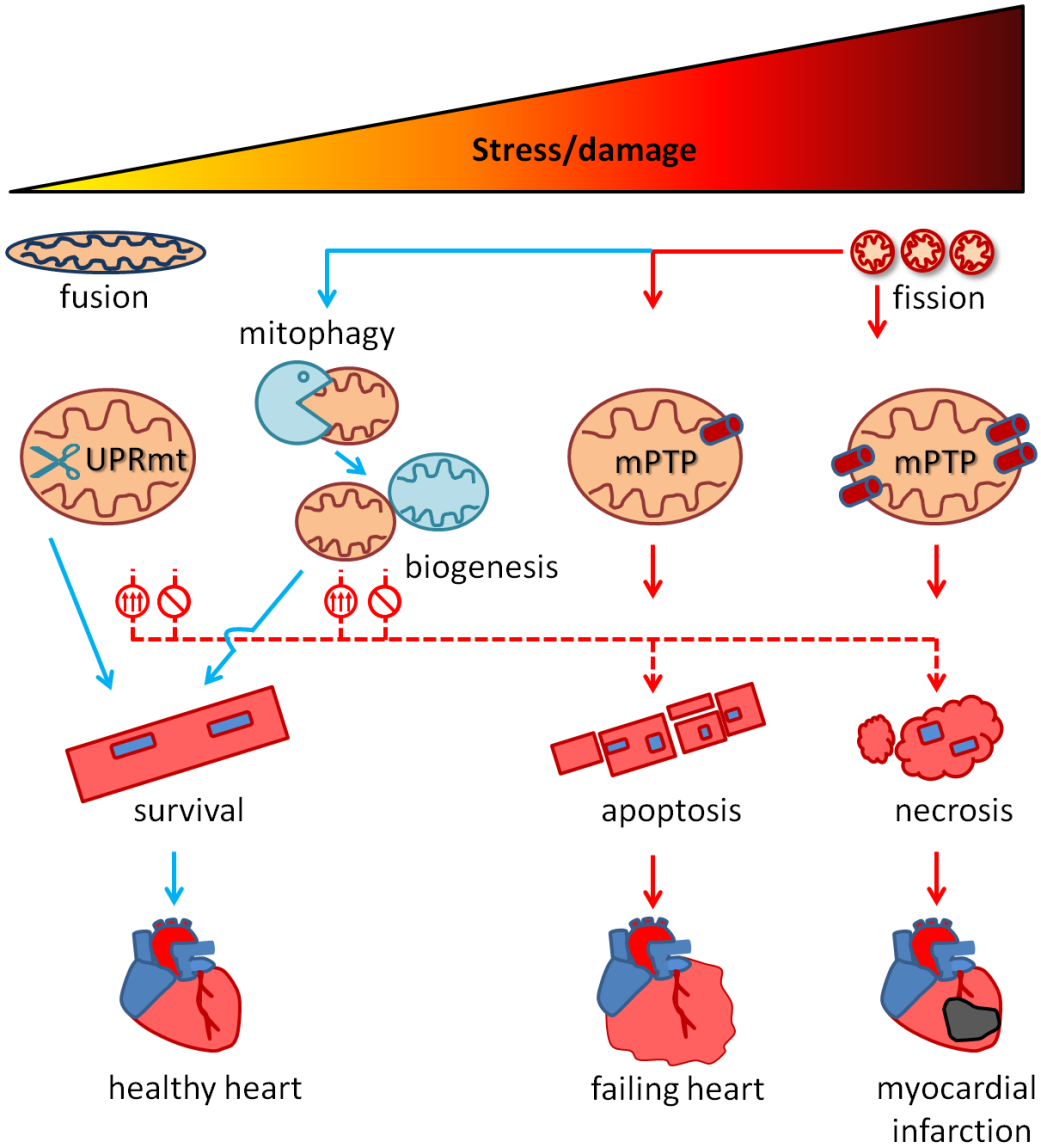
Mitochondrial quality control (MQC) in heart disease. Everyday moderate stress/damage to mitochondria is repaired by MQC mechanisms (blue arrow), which prevent the occurrence of dysfunctional mitochondria that may exacerbate stress/damage. Less extensive damage is repaired by mitochondrial unfolded protein response (UPRmt), which properly folds misfolded proteins (chaperons) or cleaves them (proteases). Mitochondria that are beyond repair undergo mitophagy, which is tightly associated with mitochondrial biogenesis, serving to maintain a pool of healthy mitochondria. Mitochondrial fusion is beneficial as it reduces reactive oxygen species (ROS) generation. Mitochondrial fission promotes healthy phenotype when allowing mitophagy. Extreme stress beyond compensation by MQC systems induces mitochondrial permeability transition pore (mPTP) opening and cardiomyocytes death (solid red arrows). Less pronounced mPTP opening allows cell death by apoptosis, which is dominant in heart failure. The most extensive cell stress induces widespread mPTP opening in cardiomyocytes, which leads to necrotic cell death, a predominant mechanism in acute ischemia-reperfusion, ie, myocardial infarction. Extremely high cell stresses are linked to mitochondrial fission, which exacerbates ROS generation. Inadequate MQC system activation induces cell death even during exposure to less extensive cell stress (red dotted arrows). This includes excessive activation of either UPRmt, mitophagy, or mitochondrial fission, which cause further dysfunction of mitochondria, or insufficient activation of MQC mechanisms that do not repair/eliminate dysfunctional mitochondria.

UPRmt is evolutionally a highly conserved MQC mechanism that helps maintain normal mitochondrial function under pathological conditions (Figure 1). It can be triggered by damage to mitochondrial proteins, imbalance between mitochondrial and nuclear proteome (mitonuclear imbalance), or other stressors, such as mitochondrial depolarization. UPRmt and endoplasmic reticulum UPR share some elements, especially transcription factors such as C/EBP homologous protein (CHOP), CCAAT/enhancer-binding protein β (C/EBPβ), or eukaryotic initiation factor 2α (eIF2α) (14,15). UPRmt involves a complex machinery of signaling molecules, transcription factors, proteases (OMI/HTRA2, lon peptidase 1 [LONP1], caseinolytic mitochondrial matrix peptidase proteolytic subunit [ClpP], paraplegin, YME1 like 1 ATPase [YME1L], mitochondrial-processing peptidase [MPP], and OMA1 zinc metallopeptidase [OMA1]), antioxidants (thioredoxin 2), endonuclease G, and chaperons (mitochondrial 70kDa heat shock protein, heat shock protein family D [Hsp60] member 1, heat shock protein family E [Hsp10] member 1, and DnaJ [Hsp40] homologue, subfamily A, member 3) (16-19). The peptides obtained by mitochondrial proteases are extruded from mitochondria by HAlF transporter (ATP Binding Cassette Subfamily B Member 10 in mammals), further activating transcription factors CHOP, C/EBPβ, and activating transcription factor 4 (ATF-4) via c-Jun/AP-1 (14,15).

One of the important mechanisms that may regulate UPRmt consists of mutually-dependent activity of two antagonistic mitochondrial proteases, YME1L and OMA1. Human ATP-dependent YME1L is an orthologue of the YME1 subunit of the yeast i-AAA complex, and OMA1 is a zinc metallopeptidase. Depending on the conditions within the cell, the two proteases can cleave and inactivate each other (20). YME1L up-regulation will result in OMA1 inactivation, and *vice versa*. Both YME1L and OMA1 can be up-regulated by stress. Constitutively active YME1L is further activated by mitochondrial depolarization with preserved ATP levels, while OMA1, which is quiescent in non-stressed cells, is activated by mitochondrial depolarization with ATP loss (20). In addition, YME1L may also be inactivated by high oxidative stress, which promotes cell death (21). Although the majority of studies indicate a negative effect of Oma1 on mitochondrial function and cellular viability, Bohovych (22) showed that OMA1 deletion impeded the stability of respiratory chain supercomplexes and mitochondrial bioenergetics in mouse embryonic fibroblasts. In the active state, YME1L triggers UPRmt by cleaving mitochondrial proteins and creating mitonuclear protein imbalance (23). The loss of activity of YME1L impairs normal mitochondrial fusion-fission dynamic, which is associated with an increase in ROS generation and increase in sensitivity to oxidative stress. This is caused by the up-regulation of OMA1 and OMA1-induced cleavage/inactivation of pro-fusion OPA1 (24). The loss of YME1L decreases cell proliferation, diminishes resistance to apoptosis, and increases protein carbonylation (16).

The beneficial effects of UPRmt are reflected in the preservation of ATP production, attenuation of excess mitochondrial ROS emission, and prevention of release/activation of mitochondrial pro-apoptotic factors (25). UPRmt-inducing transcription factor ATFS-1 promotes the assembly of oxidative phosphorylation components during mitochondrial stress, which can preserve ATP production (26). At the same time, UPRmt proteases, such as YME1L (16) or ClpP (17), can cleave dysfunctional respiratory chain components. Thus, UPRmt promotes respiratory chain recovery and helps maintaining oxidative phosphorylation in stressed mitochondria via ATFS-1 actions. However, ClpP hyperactivation by ONC201 (imipiridone) treatment diminishes oxidative phosphorylation and induces cell death in cancer cells (17).

### Mitochondrial unfolded protein response in heart disease

UPRmt is protective in chronic (27) and acute cardiac injury (18). However and seemingly contradictory, studies have also found that blocking several UPRmt elements can reduce the signs of HF of a different etiology.

One of key breakthroughs delineating the role of mitochondria in HF was a recent publication by Wai et al, who showed that cardiac-specific YME1L deletion caused HF and premature death of mice (28). An additional OMA1 deletion restored normal mitochondrial morphology and rescued the mice from HF and premature death. However, OMA1 deletion was also shown to cause developmental heart defects (22), indicating the importance of fine tuning of UPRmt effector proteases for cardiac viability. The activity of another component of UPRmt, LONP1, reduced by oxidative stress leads to the accumulation of dysfunctional respiratory chain subunits and left ventricle contractile dysfunction (29). On the other hand, LONP1 up-regulation protects cardiomyocytes from ischemia-reperfusion (I/R) injury (18). Moreover, the down-regulation of endonuclease G contributes to ROS overproduction, reduces mitochondrial DNA replication, and induces cardiac hypertrophy in rodents (19).

Conversely, there is evidence that UPRmt can be associated with harmful events in the heart. Physical exercise reduces CCAAT/enhancer-binding protein β expression in mice, and its reduction is associated with neonatal cardiomyocyte proliferation and improved resistance to pressure-induced hypertrophy (14). Furthermore, excessive eIF2α activation via double-stranded RNA-dependent protein kinase (PKR) promotes cardiomyocyte apoptosis and HF (30). Parvostatin blocks UPRmt activator c-Jun, which improves left ventricular function and slows the progression of HF in mice (31). Moreover, elevated LONP1 activity is a mediator of hypoxia-induced cardiomyocyte apoptosis and its down-regulation attenuates ROS generation and protects the cells (32), while ClpP deletion increases the expression of respiratory chain subunits and reduces cardiomyopathy (33). In the aged rat heart, the elevated activity of OMI/HTRA2 protease promotes mitochondrial depolarization and apoptosis (34), and its overexpression causes apoptosis and cardiac dysfunction in transgenic mice (35). Gain-of-function mutation of paraplegin (SPG7) increases mitochondrial ROS generation and coronary artery disease risk (36). Lin et al (37) linked high HSP60 expression to proinflammatory state and cardiomyocyte damage in human HF. Elevated expression/activity of several UPRmt elements was demonstrated in humans and animals with HF, including LON and ClpP (38), CHOP (15), eIF2α (15), ATF-4 (39), and c-Jun N-terminal kinases (40).

A potential explanation for such seemingly contradictory findings is that UPRmt is cardioprotective when moderately active, while its excessive activity may be cardiotoxic. A moderate activation of UPRmt may be beneficial for removing/repairing damaged mitochondrial proteins and thereby maintaining normal mitochondrial and cardiac function. An excessive UPRmt activation could result in a massive cleavage of mitochondrial proteins, exacerbating mitochondrial dysfunction and promoting heart damage. It is difficult to discriminate cardiotoxic from cardioprotective actions of UPRmt in the absence of reliable pharmacological inhibitors/activators and using only gene overexpression/knockouts models, which are not well suited for testing fine dose-responses.

## Mitophagy

Mitochondrial damage with the loss of polarity on the outer mitochondrial membrane (OMM) is a signal for the removal of mitochondria by mitochondrial autophagy (mitophagy). The complex processes involved in mitophagy are in more detail described elsewhere (3). Mitochondrial fragmentation is essential for mitophagy, whereby fission enables the sequestration of damaged mitochondrial parts into a mitochondrion, which is eliminated by mitophagy. Crucial molecular regulators of mitophagy are Parkin (E3 ubiquitin ligase) and PTEN-induced putative kinase protein 1 (PINK1) (41,42). Under normal conditions, when mitophagy is not occurring, Parkin is self-ubiquitinated and inactivated and PINK1 is degraded (3,24). PINK1 is degraded by MPP and presenilin-associated rhomboid-like (PARL) after it is imported into the mitochondrial matrix via the translocase of the outer membrane 40 and translocase of the inner membrane 23 complexes (24). The loss of mitochondrial membrane potential prevents the import of PINK1, which then accumulates in the TOM complex. PINK1 autophosphorylates and activates itself and phosphorylates and activates Parkin. Parkin ubiquitinates mitochondrial OMM proteins and thereby targets them for phagosomal digestion. In the process of mitophagy, Parkin also interacts with PRKR-like endoplasmic reticulum kinase, ATF-4, MFN2, mitochondrial rho GTPase 1, voltage-dependent anion-selective channel 1, and hexokinase-1 (3,39,43). The mitochondrion is engulfed by the autophagosome, which eventually fuses with lysosome for digestion. The autophagosome formation depends on the proteins optineurin, nuclear domain 10 protein 52, sequestosome 1 (p62), microtubule-associated proteins 1A/1B light chain 3B (LC3), and TANK-binding kinase 1 (3,42). The phagophore membrane elongation is conveyed by proteins from the autophagy-related genes (ATG) family. An important factor in autophagosome formation is unc-51 like autophagy activating kinase 1 complex. It phosphorylates a large number of mitophagy proteins, such as Beclin-1, p62 and FUN14 domain containing 1 (FUNDC1) (5). The autophagosome maturation and fusion with lysosome is mediated by Ras-associated protein 7, TBC1 domain family member 15 (TBC1D15), TBC1D17, homotypic fusion and protein sorting complex, pleckstrin homology and RUN domain containing M1, syntaxin-17, and vesicle-associated membrane protein 8 (3). Parkin-independent mitophagy occurs via BCL2 interacting protein (BNIP3), NIP3-like protein X (NIX), FUNDC1, BCL2 like 13, GABA(A) receptor-associated protein (GABARAP) and GABARAPL1 (5).

### Mitophagy in heart disease

Mitophagy is crucial for heart function and development. The interruption of PINK1/MFN2/Parkin pathway in mice causes lethal cardiomyopathy by 7-8 weeks, while the surviving mice exhibit abnormal energy metabolism in the heart (43).

Similarly to UPRmt, there is a fine line between protective and deleterious effects of mitophagy in cardiomyocytes. BNIP3 is transcriptionally up-regulated in the heart by hypoxia, whereas myocardial NIX up-regulation appears to be a specific transcriptional response to pathological hypertrophy (44). Forced NIX expression in cardiomyocytes leads to progressive apoptotic cardiomyopathy and premature death (45). The ablation of BNIP3 reduces apoptosis in ischemic cardiomyocytes (46). A very similar effect in heart remodeling is observed in Nix knockout mice, which were protected from ventricular dilation, wall thinning, and systolic dysfunction following heart pressure overload (45). Conversely, Nix and Bnip3 double knockouts develop cardiomyopathy with reduced ejection function of the left ventricle. This indicates that both NIX and BNIP3 may play a detrimental and beneficial role in the heart, depending on the (patho)physiological context and perhaps the extent of their activity.

Hypoxia activates FUNDC1, which interacts with LC3, thereby, activating mitophagy in platelets, diminishing their activity, which ameliorates cardiac reperfusion injury (47). The reduced levels of FUNDC1 (5) and PINK1 (41) are found in human samples of HF. Conversely, other studies show an elevated expression of mitophagy markers, including Parkin and LC3-mediated formation of autophagosome (42) and BNIP3 (48), in human and animal samples of HF of different etiologies. Cardiac-specific Fundc1 knockout mice exhibit impaired cardiac function, the accumulation of elongated and dysfunctional mitochondria and a greater degree of MI-induced HF (5). Similarly, PINK1-deficient mice are more susceptible to pressure overload-induced HF (41) and I/R heart injury (49). Moreover, the stimulation of PINK1/Parkin-mediated mitophagy by AMP-activated protein kinase α2 (AMPKα2) overexpression protects from pressure-induced HF (50). Parkin overexpression protects the heart from aging-induced dysfunction and cell senescence (51).

Mitochondrial function is unaffected in Parkin deficiency, although such mitochondria seem smaller, with a disorganized network (52). However, Parkin-deficient mice are more sensitive to I/R injury, which can be reduced by Parkin overexpression in isolated cardiac myocytes (52). Moreover, ATG5 depletion induces HF (53), while ATG7 induction may ameliorate desmin-related cardiomyopathy (54). Impaired mitochondrial fusion-fission balance (55) and AMPKα2 expression (50,56) may lead to cardiomyopathy and/or cardiomyocyte necrosis in part by disrupting mitophagy. Pharmacological and non-pharmacological mitophagy inducers, such as rapamycin, mainly reduce cardiac I/R injury or pathological cardiac remodeling and HF (57).

Overall, similarly to UPRmt, studies have showed positive and negative effects of mitophagy in heart disease, which could be related to the extent of mitophagy activation.

## Mitochondrial biogenesis

Mitochondrial biogenesis is described in detail in the review by Ploumi et al (58). In brief, mitochondrial biogenesis is driven to a lesser extent by mitochondrial DNA (mtDNA) and to a greater extent by nuclear DNA that harbors essential regulatory processes. The key factors of mtDNA replication include mitochondrial DNA polymerase γ (POLG) and twinkle (resembles helicase) (59,60). POLG, mitochondrial transcription factor A (TFAM), and adenine nucleotide translocase (ANT) are important for mtDNA maintenance and repair (61-63). Mitochondrial DNA transcription elongation factor defines whether replication or transcription takes place. The transcription initiation complex consists of TFAM, mitochondrial DNA-directed RNA polymerase, and mitochondrial dimethyladenosine transferase 2 enzyme (58). These transcription factors and coactivators regulate other processes, such as respiratory chain assembly or fatty acid oxidation, and their impairment can affect cardiac function in multiple ways.

Nuclear respiratory factors 1 and 2 (NRF1 and NRF2) are transcription factors that regulate the expression of numerous genes involved in mitochondrial assembly (64,65). NRF2 is negatively regulated by cytoplasmic protein KEAP1, which marks it for degradation by ubiquitination (58). The expression of genes involved in mitochondrial metabolism is controlled by estrogen related receptors (ERR)α and ERRγ, whereby ERRα activates peroxisome proliferator activated receptor (PPAR)α and up-regulates NRF1 (66-68). The central role in the regulation of mitochondrial biogenesis belongs to PGC-1α. It is a transcription coactivator of PPARα, PPARβ, PPARγ, ERRα, ERRβ, ERRγ, NRF1 and NRF2 (69). A similar role is played by PGC-1β (69).

### Mitochondrial biogenesis in heart disease

An impaired mitochondrial biogenesis leads to HF and increases the sensitivity to MI. A factor that interferes with mitochondrial biogenesis is ANT deficiency, which destabilizes mtDNA, increases ROS production, and causes HF (63). Cardiac-specific Tfam knockout mice display reduced ATP generation, increased apoptosis, atrioventricular conduction block, and dilated cardiomyopathy (70-72). Conversely, TFAM overexpression protects the heart from MI-induced HF (62). Similarly, the overexpression of TFAM or twinkle improves cardiac function in a pressure or volume overload and reduces ROS overproduction (59,60). Poor mtDNA replication and accumulation of mutations, induced by inactive POLG, leads to dilated cardiomyopathy and interstitial fibrosis (61).

NRF1- or NRF2-deficient mice exhibit low levels of mtDNA, left ventricular dysfunction, and die before birth (64,65). Conversely, NRF2 induction by preconditioning or pharmacological treatment protects against MI (73). ERRα-deficient mice exhibit cardiac dysfunction only after pressure overload (66), while ERRγ deficient mice exhibit lethal HF (67). The concomitant deletion of ERRα and ERRγ leads to prenatal death (74). While mice with prenatal knockout of either Pgc-1α or Pgc-1β experience mild cardiac dysfunction only after the exposure to noxious stimuli (75,76), prenatal knockouts of both proteins cause HF and prenatal death (69), indicating an overlap in their function. On the other hand, their deletion in adulthood is not associated with significant heart dysfunction (77). The same study also demonstrated that PGC-1α and PGC-1β deficiency decreases the expression of pro-fusion factors MFN2 and OPA1, and pro-fission factor fission 1 (FIS1), leading to abnormal mitochondrial morphology. This further shows multiple interplays among different MQC mechanisms. PGC-1α-induced biogenesis may act beneficially by reducing mitochondrial calcium uptake (78), which is a powerful stressor and inducer of mPTP opening (1,79). Mitochondrial permeability transition pore opening is induced by ROS and mitochondrial calcium overload and precedes cardiomyocyte death (80,81). However, in dilated (and not ischemic) cardiomyopathy, mitochondrial biogenesis is enhanced (increased expression of PGC-1α, POLG, POLG2, and TFAM) (82). This possibly reflects a compensatory response to mitochondrial dysfunction arising from mtDNA mutations that were the most prevalent in dilated cardiomyopathy (82). Conversely, in samples of HF with reduced ejection fraction, PGC-1α levels are reduced together with complex IV activity (48), which may suggest that poor biogenesis contributes to cardiac dysfunction.

Altogether, impaired mitochondrial biogenesis predisposes to HF and increases the extent of MI, while enhanced expression of biogenesis genes may correspond to compensatory response to mitochondrial dysfunction.

## Mitochondrial fusion-fission balance

Mitochondria constantly undergo fusion and fission. Mitochondrial dynamics is associated with the metabolic state of the cell, presence of various stressors, and other important processes, such as cell proliferation and differentiation. Mitochondrial fusion-fission is reviewed in detail by Ježek et al (4). Fusion-fission dynamics work tightly with other MQC mechanisms, UPRmt, mitophagy, and biogenesis.

The key protein responsible for mitochondrial fission is GTP-ase dynamin-related protein 1 (Drp1) (83). Other pro-fission factors that interact with Drp1 and promote mitochondrial fission include FIS1 and mitochondrial dynamics proteins mitochondrial dynamics protein of 49 KDa (MID49) and MID51 (4,84). Once activated, Drp1 translocates from the cytoplasm onto mitochondria causing fission, which also requires the action of dynamin-2 (85). The mitochondrial fusion depends on three GTP-ases, MFN1, MFN2, and OPA1 (86,87). While MFN1 and MFN2 mediate OMM fusion, OPA1 mediates inner mitochondrial membrane fusion. In addition to regulating the morphology of mitochondrial cristae, OPA1 also prevents apoptosis in physiological conditions, while pro-fission factors, such as Drp1, promote apoptosis (4,86,88). OMA1 and YME1L proteases are key regulators of OPA1 activity, whereby OMA1 cleaves and inactivates it (88).

### Mitochondrial fusion-fission balance in heart disease

Extensive fission (fragmentation) of mitochondria has been associated with cardiac I/R injury (84,89), which is caused by enhanced FIS1 and Drp1 expression in mitochondria and/or reduced MFN1 and OPA1 expression (90). OPA1 deficiency reduces mtDNA copy number, decreases mitochondrial and heart function, and leads to cardiomyopathy (91). Conversely, the induction of mitochondrial fusion by OPA1 overexpression protects cardiac (92) and other cell types (93) from various types of stressors. Zaja et al (83) showed that I/R injury induced mitochondrial fission, cardiomyocyte death and Drp1 activation (phosphorylation of Ser616 and dephosphorylation of Ser637), while it did not significantly change the expression of MFN1, MFN2 and OPA1. Drp1 activation results in cardiomyocyte death, with ROS and calcineurin acting as upstream modulators of Drp1 activity (83). Pharmacological Drp1 inhibition with mdivi-1 or calcineurin inhibition with FK506 reduces mitochondrial fission and cell death (83). Ong et al (84) have demonstrated that both the transfection of HL-1 cells with the Mfn1, Mfn2, or a dominant-negative mutant form of Drp1 (Drp1K38A), or pharmacological Drp1 inhibition, promote mitochondrial elongation and reduce mPTP opening and cell death after simulated I/R injury. They also showed that human FIS1 overexpression reduced mitochondrial elongation, increased cell death, but without an effect on mPTP opening. Disatnik et al (89) used selective Drp1 inhibitor (P110) in *ex vivo* and *in vivo* rat heart model of MI to assess the role of Drp1/FIS1 interaction in reperfusion injury. They found that increased mitochondrial fragmentation during reperfusion facilitated long-term cardiac dysfunction in rats, whereas acute inhibition of mitochondrial fission early at the onset of reperfusion resulted in long-term benefits. Conversely, FUNDC1 ablation promoted mitochondrial elongation via FIS1 down-regulation, which caused mitochondrial dysfunction and HF in mice (5). Moreover, FUNDC1 and FIS1 are down-regulated and mitochondria are elongated in HF samples (5). This suggests that the absence of mitochondrial fission and predominance of mitochondrial elongation (fusion) could be detrimental to the heart. However, this is inconsistent with the majority of other studies, which show an increase in mitochondrial fission in different types of HF and cardiomyocyte injury (48,82,86). The expression of pro-fusion and pro-fission proteins varies among studies and types of HF. Ahuja et al (82) and Chen et al (86) found OPA1 decrease in ischemic heart and MFN2 increase in dilated cardiomyopathy. Conversely, these two studies found an opposite change in MFN2 in ischemic cardiomyopathy. In the first study it was decreased and in the second it was increased. In dilated cardiomyopathy, Ahuja et al found increased OPA1 and Chen et al found no change in OPA1. The latter study also found an increase in MFN1 and Drp1 and no change in FIS1 in both types of cardiomyopathy (86). Increased Drp1 expression was also found in the samples of HF with reduced ejection fraction (48). HF of different etiology in animal models is associated with increased mitochondrial fragmentation, decrease in pro-fusion factors, and increase in pro-fission factors: OPA1, MFN1, and Drp1 (94); OPA1, MFN1, MFN2, Drp1, and FIS1 (87); MFN2 and Drp1 (95) and MFN2 (Drp1 was reduced) (42). In the majority of these studies, mitochondrial fragmentation was associated with elevated ROS generation, diminished ATP production, and cardiac dysfunction/damage, while the shift toward mitochondrial fusion exhibited beneficial effects. Along these lines, Drp1 inhibition by mdivi-1 ameliorates the signs of HF arising from pressure overload (96). However, Drp1 is important for cardiac function. Its mutation causes HF due to impaired mitophagy, mitochondrial depolarization, poor calcium handling, and diminished ATP synthesis (97).

The majority of studies demonstrate beneficial effects of mitochondrial fusion and detrimental effects of fission in acute cardiac I/R injury, suggesting that mitochondrial fusion increases the resistance to cell death possibly by attenuating ROS generation and preserving ATP production. In chronic HF experiments, some of the results reported are not congruent with those by other investigators. Inconsistencies in results among studies testing human HF samples may arise from different reasons, such as methodological issues, biological variability of individuals/populations, and different etiologies of HF. Thus, a fusion-fission imbalance can lead to or accelerate HF, reflecting the importance of both states for long term mitochondrial and cardiac function.

## Mitochondrial quality control in the aged heart

Aging is associated with a decline in cardiac function and increased incidence of MI and HF. Aging impairs mitochondrial function in part by dysregulation of MQC (98). Limited perturbations of mitochondrial function can induce UPRmt, a process that is associated with lifespan extension (99). Conversely, there are studies that demonstrate that triggering of UPRmt may reduce lifespan (99). UPRmt protease LON is down-regulated in aged cells (100). However, there is a lack of studies investigating UPRmt in the aged heart. The overexpression of ATG5 protein may extend lifespan (101), and its inhibition could lead to age-related cardiomyopathy in mice (102), suggesting that impairment of autophagy/mitophagy could contribute to degenerative changes in the aged heart. Parallel ablation of MFN1, MFN2, and Drp1, which impaired mitochondrial dynamics, causes “mitochondrial senescence” and cardiac changes that resemble aging-related cardiomyopathy (103). In agreement to this study, Zhao et al found a reduced expression of p62, LC3-IIm PGC-1α and MFN2 in the aged (25-month-old) rat heart, indicating reduced autophagy/mitophagy, mitochondrial biogenesis and fusion (104). Using 24-month-old mice, hearts Whitehead et al also found a reduced expression of MQC genes: PGC-1α, PGC-1β, TFAM, ERRα, MFN1, MFN2 and OPA1 but observed no change in FIS1 and Drp1 (105). Conversely, another study with older rats (36-months-old) showed elevated cardiac Drp1 and OPA1 expression (106).

Most of the existing studies suggest that the MQC is reduced in the aged heart and that its down-regulation shortens lifespan and leads to phenotypes found in the aged heart. However, there are data showing that some MQC components may be up-regulated in the aged heart. It is possible that, as in heart disease, MQC could exert beneficial or maybe even detrimental effects in the aged heart. More studies are required to resolve such seemingly conflicting data.

## Conclusions

MQC consists of several interconnected mechanisms that serve to maintain proper mitochondrial function. Numerous studies demonstrate that failure in some of MQC mechanisms may induce HF or exacerbate MI, and that stimulation of MQC attenuates cardiac injury/dysfunction. On the contrary, a large number of studies also show that MQC elements are elevated in HF and that MQC down-regulation has protective effects on the heart. It is possible that the effects of MQC on mitochondrial and cardiac functions are nonlinear or biphasic (107). In other words, a moderate activity of MQC may improve overall mitochondrial function and be beneficial for the heart as a compensatory response. However, hyperactive MQC could lead to excessive perturbation of mitochondrial homeostasis (eg, excessive mitochondrial removal or protein cleavage), leading to cardiac injury or exacerbating the existing pathological processes.
